# Family Impacts of Severe Dental Caries among Children in the United Kingdom

**DOI:** 10.3390/ijerph17010109

**Published:** 2019-12-22

**Authors:** Rawan Abed, Eduardo Bernabe, Wael Sabbah

**Affiliations:** 1 Eastman Dental Institute, University College London, 256 Grays Inn Rd, London WC1E 6DG, UK; rawan.abed.19@ucl.ac.uk; 2 Faculty of Dentistry, Oral & Craniofacial Sciences, King’s College London, Bessemer Road, London SE5 9RS, UK; wael.sabbah@kcl.ac.uk

**Keywords:** schoolchildren, dental caries, quality of life, family

## Abstract

The aim of this study was to evaluate the family impacts of severe dental caries among children. Data from 3859 school-age children (5-, 8-, 12- and 15-year-olds) who participated in the 2013 Children’s Dental Health Survey, a national cross-sectional survey in England, Wales and Northern Ireland, were used. Severe dental caries was defined as having at least one tooth with pulpal involvement, ulceration, fistula, or abscess (PUFA). Family impacts were measured using seven items of the Family Impact Scale (FIS). The association between severe dental caries and family impacts was assessed in logistic regression models, adjusting for child’s age, gender, and country of residence; parent’s marital status, education, and job classification; and area deprivation. Severe dental caries among children showed a significant negative impact on family life (Odds Ratio: 6.00; 95% Confidence Interval: 3.34–10.78). Parents of children with severe dental caries had greater odds of taking time off work (OR: 2.75; 95% CI: 1.16–6.54), reporting the child needed more attention (OR: 4.08; 95% CI: 2.15–7.75), feeling guilty (OR: 6.32; 95% CI: 3.26–12.26), feeling stressed (OR: 7.34; 95% CI: 4.15–12.99), having normal activities disrupted (OR: 5.78; 95% CI: 2.71–12.34), and having sleep disrupted (OR: 4.94; 95% CI: 2.78–8.76). Having severe dental caries was not associated with financial difficulties in the family (OR: 1.64; 95% CI: 0.49–5.51). The observed association between severe dental caries and family impacts was independent of child and family sociodemographic characteristics. The findings underscore the importance of preventive interventions to avoid severe dental caries in children and subsequently reduce negative impacts on their family life.

## 1. Introduction

Dental caries is the most common oral condition in childhood [1,2]. The World Health Organization (WHO) reported that dental caries affects around 60–90% of children at school age, which negatively affects the quality of life of children and their families [3]. Moreover, poor socioeconomic circumstances of the family exacerbate the development and progress of dental caries [4,5,6,7].

Most epidemiological surveys around the world use a standard method for caries assessment [8], which is based on either the WHO criteria [9] or the International Caries Detection and Assessment System (ICDAS) [10]. However, these diagnostic criteria do not accurately reflect the consequences of untreated dental caries [11]. The count of teeth with pulpal involvement, ulcers, fistula, or abscess (PUFA) has been recommended to record the sequelae of dental caries when left untreated [11,12]. While this count is usually high in deprived countries, it is not uncommon in rich countries, such as the United Kingdom (UK), particularly among deprived communities [13]. 

Childhood dental caries, when left untreated, is usually associated with dental pain and inability to eat or sleep [14,15,16,17], which in turn can negatively impact the child’s daily activities, including school attendance [18,19]. Several studies have shown that untreated caries among children can have a negative impact on their families as parents feel upset and guilty, take time off work to take care of the child, and endure financial difficulties [20,21,22,23,24,25,26]. However, most of these studies did not account for important confounders during analysis, such as the family structure [20,21,22,23,24,25,26] and socioeconomic circumstances [23,24,25,26], which are known determinants of both childhood dental caries and family life. Furthermore, most studies that examined the relationship between dental caries and impacts on family used the count of decayed, missing, and filled teeth (DMFT index). However, to the best of our knowledge, no study has assessed the impact of severe dental caries, which extends to the pulp or the periapical area of the tooth, on family life. As having teeth with PUFA is considered more accurate for detecting severe dental caries than using the DMFT index [11], it was hypothesized that using PUFA would provide a better explanation for the relationship with family impacts. In other words, parents are more likely to have their sleep disturbed or social activities interrupted if the child suffers from severe pain or infection rather than just having an asymptomatic dental problem. Therefore, the aim of this study was to evaluate the family impacts of severe dental caries among children in the UK.

## 2. Materials and Methods 

This study was based on secondary analysis of the 2013 Children’s Dental Health Survey (CDHS). The survey protocol was approved by the ethics committee at University College London (ID: 2000/003). Written consent was provided by parents, but children could opt out at the time of the examination.

### 2.1. Data Source

**Figure 1:**
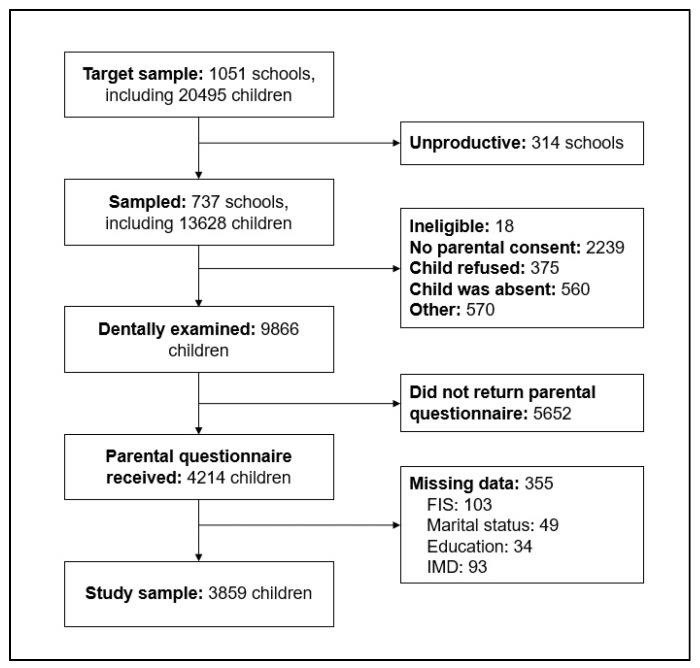
Participation at different stages of the 2013 Children’s Dental Health Survey. FIS: Family impact scale. IMD: index of multiple deprivation.

The 2013 CDHS is a nationally representative cross-sectional survey of 5-, 8-, 12- and 15-year-old children in England, Wales, and Northern Ireland. Participants were recruited via multi-stage stratified sampling. Information was collected via parental questionnaires and child dental examinations. The number of participants in the survey who had clinical assessment was 2549 5-year-olds, 2367 8-year-olds, 2532 12-year-olds, and 2418 15-year-olds [27]. Of the 9866 children who were clinically examined, 4214 had completed parental questionnaires (which included information on family impacts and socioeconomic position). Of these, 355 were excluded because of missing values on other relevant variables. The current analysis included 3859 participants who had complete data on dental status, family impacts, and sociodemographic characteristics (Figure 1).

### 2.2. Selection of Variables

The outcome variable in this study was the family impacts related to children’s oral health. The survey assessed the impacts on family life using items from the 14-item Family Impact Scale (FIS) [28] of the Parental-Caregiver Perceptions Questionnaires (P-CPQ) [29]. The FIS includes 5 items on parental/family activity, 4 on parental emotions, 4 on family conflict, and 1 on financial burden. Due to competing demand for space and concerns about completion times [27], the parental questionnaire of the CDHS only included seven questions, which related to impacts in the last 6 months on the following domains: parental/family activity domain: took time off work because of the child’s dental health, the child needed more attention, parent’s sleep was disturbed, and normal activities were interrupted; parental emotion domain: parents felt guilty, and parents felt stressed or anxious, because of the child’s dental health; financial burden domain: financial difficulties. Responses were given using five options, namely: never, once or twice, sometimes, often, and every or almost every day. Due to the low prevalence of impacts, items were dichotomized for analysis (never versus once or more often). They were analyzed individually as well as jointly (no impact versus any impact). The FIS has been used in the UK previously [30]. The Cronbach’s alpha was 0.78 in this sample.

Severe dental caries was the main exposure, which was determined during clinical examinations and recorded as the number of teeth with pulp involvement, ulceration, fistula, or abscess (PUFA). Each clinical finding was recorded into 3 groups: 0, 1, and 2 or more lesions. A dichotomous variable indicating whether the child had any tooth with PUFA was created. Clinical examinations were conducted by 75 dentists, who were trained and calibrated in 8 groups. Intra-examiner reliability for tooth condition was high (Kappa values ranged from 0.814 to 0.928) [27].

The analysis included several child and family characteristics that might confound the association between severe dental caries and family impacts. Child characteristics were gender, age (5, 8, 12, and 15 years) and country of residence (England, Wales, and Northern Ireland). Family characteristics were the parent’s marital status (living with partner or alone), education (no qualification, below degree level and at degree level or above) and job classification (managerial and professional, intermediate, routine or manual, and never worked), and the quintile of deprivation of the residence area (based on the household’s postcode) according to the index of multiple deprivation (IMD).

### 2.3. Data Analysis 

All analyses were conducted using Stata 15 (StataCorp., College Station, TX, USA). To account for the complexity of the survey, parent’s questionnaire weights and survey features (stratification and clustering) were used throughout the analysis. We first compared the prevalence of severe dental caries by child and family sociodemographic characteristics with the Chi-squared test. 

The association between severe dental caries among children and any impacts on family life was tested in logistic regression models. Odds ratios (OR) were the associational measure reported. They were adjusted for child demographic characteristics (gender, age, and country of residence) and family sociodemographic characteristics (parent’s marital status, education, job classification, and IMD quintiles). Separate regression models were also reported for the association between severe dental caries and each of the seven FIS items (time off work, financial difficulties, child needed more attention, parent’s sleep disrupted, normal activities disrupted, parent felt guilty, and parent felt stressed). The interaction between child gender and age was added to each regression model for statistical testing. The conventional significance level of 0.05 was used for hypothesis testing.

## 3. Results

Table 1 exhibits the sociodemographic characteristics of the study sample. Almost thirty percent of parents (29.5%) reported impacts on family life due to their child’s oral health, with the most frequent family impacts relating to taking time off work (15.5%), feeling stressed (14.2%), child needing more attention (13.4%), and feeling guilty (10.4%), and the least frequent impacts relating to financial difficulties (2.4%), normal activities disrupted (6.1%), and sleep disrupted (7.4%). There were significant differences in the prevalence of severe dental caries by parent’s job classification but not by parent’s education or IMD quintiles (Table 1). The prevalence of severe dental caries was the highest among 8-year-olds, but there were no differences by gender or country of residence. 

Table 2 exhibits the distribution of any family impacts within all explanatory variables. Most parents of children with severe dental caries reported an impact on their lives (66.2%). Furthermore, the highest percentages of impact on family life were reported by parents with routine and manual jobs, no educational qualification, and those who lived in the most deprived areas. In the adjusted regression model, only severe dental caries and child age were significantly associated with any family impacts. Parents of children with severe dental caries had 6.00 (95% CI: 3.34–10.78) times greater odds of reporting impacts on family life than those of children without such a condition. Parents of older children had greater odds of reporting family impacts. 

The results from the adjusted regression models for each of the seven items on family impact are shown in Table 3. Accounting for child and family sociodemographic characteristics, parents of children with severe dental caries had greater odds of reporting taking time off work (OR: 2.75; 95% CI: 1.16–6.54), the child needing more attention (OR: 4.08; 95% CI: 2.15–7.75), disruption of the parent’s sleep (OR: 4.94; 95% CI: 2.78–8.76), disruption of normal family activities (OR: 5.78; 95% CI: 2.71–12.34), feeling guilty (OR: 6.32; 95% CI: 3.26–12.26), and feeling stressed (OR: 7.34; 95% CI: 4.15–12.99). The only item that was not associated with severe dental caries among children was financial difficulties (OR: 1.64; 95% CI: 0.49–5.51). There were no significant associations between any of the family socioeconomic indicators and any family impacts. Finally, the two-way interaction between child gender and age was not significant when added to each logistic regression model (*p* < 0.05 in all cases). Therefore, it was not included in the final models reported. 

## 4. Discussion

This study assessed the family impacts of severe dental caries, indicated by any tooth with PUFA, among schoolchildren in the UK. The findings demonstrated that severe dental caries was related to negative impacts on family life. The strongest impacts of any child’s severe dental caries were related to parents feeling guilty and being stressed because of the child’s dental problem. Given the parents’ responsibilities for the child’s wellbeing, it is not surprising that perceptions of guilt and stress were the greatest implications of a child experiencing severe dental caries associated with pain and infection. Furthermore, the findings indicate that the presence of teeth diagnosed with PUFA among children can adversely impact family life by restricting their normal activities, causing them to take time off work, and disturbing their sleep, as they had to care for a child in pain. 

These findings are consistent with earlier research, which demonstrated an association between childhood caries and negative family impacts [20,21,22,23,24,25,26]. However, all earlier research used the DMFT index, which also captures mild (usually asymptomatic) disease stages. Our case definition (severe dental caries) highlighted the greater prevalence of family impacts associated with advanced forms of the disease rather than those associated with any caries experience. What is more, our findings by FIS items helped to characterize what areas of family life are often disrupted by the child’s condition. It is worth noting that in the regression models adjusting for child demographic factors and three different indicators of family socioeconomic position, severe dental caries was the only factor that showed a consistent and significant relationship with all indicators of family impacts. 

The negative impacts of children’s severe dental caries on family life were higher among those with lower socioeconomic positions, particularly the least educated, manual or routine occupations, and living in deprived areas. However, after accounting for severe dental caries and other demographic factors, there were no statistically significant associations between socioeconomic indicators and family impacts. The only exception was for unemployed parents who had a great adverse financial impact due to children’s oral health problems. This could be attributed to the additional financial burden imposed on these families with lower income as they are trying to seek help for a child suffering severe dental caries [31]. Similar findings were reported in other studies reporting a significant relationship between financial distress and each of early caries lesions and DMFT [23,24]. 

Severe dental caries indicated by the presence of pulpal involvement and periapical infection is more common in low-income countries [11]. However, a considerable portion of UK children had at least one tooth with PUFA as indicated in this analysis. This finding is particularly shocking given the availability of free dental services to all children living in the UK under the National Health Service (NHS), thus indicating indirect barriers to the use of services, rather than the direct cost of treatment [32]. These indirect barriers, along with observed impacts on family life, which were independent of socioeconomic conditions, highlight the importance of adopting social and economic policies that aim at reducing the indirect cost of dental treatment. The findings also indicate the need for health-promoting policies aimed at changing the food environment, and subsequently changing eating habits of the children, by restricting access to unhealthy food and improving access to affordable healthy food. In other words, making the healthy choice the easy choice [33]. This could be complemented by caries preventive programs in schools (topical fluorides and fissure sealants). As for further research, this area would benefit from studies in alternative settings and younger age groups (pre-school children). Qualitative studies can shed some light on how severe dental caries among children disrupts family activities and how families cope with such problems. 

The current analysis has the advantage of using data from a nationally representative sample for England, Wales and Northern Ireland. However, there are a few limitations worth mentioning. Firstly, the cross-sectional nature of the survey does not support inference on causality or temporality between severe dental caries and family impacts. Secondly, selection bias could have arisen from two sources, either because some families did not return the parental questionnaire or because they did not answer all questions. However, the analytical weights for the parental questionnaire data were used to compensate for this limitation and make the findings generalizable to the full study population. Furthermore, there were no major sociodemographic differences between children in our study sample and those excluded because of missing data on relevant variables. Thirdly, our assessment of family impacts was prone to measurement bias because it included only seven of the 14 items in the FIS. Such misclassification of outcome is likely to be non-differential (i.e., it affected children with and without severe dental caries equally), and therefore will tend to attenuate the association between severe dental caries and family impacts in the study sample. That said, the findings observed by specific FIS items do not share this limitation and provided richer information on the specific family activities that were disrupted. 

## 5. Conclusions

Severe dental caries among children had a significant negative impact on family life, most notably on parental perceptions of stress and guilt, interruption of normal family activities, sleep disturbance and missing working days. The relationship between severe dental caries and family impacts was independent of child demographic characteristics and family socioeconomic factors. 

## Figures and Tables

**Table 1:** Description of the sample and prevalence (%) of severe dental caries among children (any tooth with PUFA) by explanatory variables (n = 3879).

Explanatory Variable		All Sample		Severe Dental Caries		*p*-Value ^a^
		n	%	%	[95% CI]	
*Gender*						0.644
	Male	1913	50.5	4.1	[2.6–6.4]	
	Female	1946	49.5	3.6	[2.3–5.7]	
*Age*						<0.001
	5-year-old	1126	25.5	4.5	[2.5–7.8]	
	8-year-old	1081	24.8	8.8	[6.1–12.5]	
	12-year-old	891	23.7	2.2	[1.1–4.2]	
	15-year-old	761	26.0	0.1	[0.02–0.7]	
*Country*						0.713
	England	2052	91.4	3.8	[2.6–5.6]	
	Wales	796	5.1	4.8	[3.0–7.6]	
	Northern Ireland	1011	3.5	3.6	[1.9–6.7]	
*Parent’s marital status*						0.144
	Living with partner	3118	79.4	3.5	[2.4–5.2]	
	Living alone	741	20.6	5.3	[3.1–8.9]	
*Parent’s education*						0.796
	At degree level or above	1424	34.6	4.1	[2.6–6.6]	
	Below degree level	2064	54.4	3.9	[2.5–5.8]	
	No qualification	371	11.0	3.1	[1.4–6.5]	
*Parent’s job classification*						0.002
	Managerial and professional	1581	39.2	1.9	[1.0–3.6]	
	Intermediate	983	24.0	7.0	[4.6–10.5]	
	Routine and manual	1000	27.5	3.3	[1.9–5.9]	
	Never worked	295	9.3	5.9	[2.7–12.4]	
*IMD quintiles*						0.294
	Most deprived (Q5)	810	27.2	5.1	[2.7–9.4]	
	Second most deprived (Q4)	806	19.6	4.2	[2.3–7.5]	
	Middle (Q3)	839	17.6	3.5	[1.9–6.4]	
	Second least deprived (Q2)	806	19.2	4.3	[1.9–9.1]	
	Least deprived (Q1)	598	16.4	1.4	[0.7–2.7]	

PUFA: pulp involvement, ulceration, fistula, or abscess; IMD: index of multiple deprivation. ^a^ Chi-squared test was used for comparisons.

**Table 2:** Regression model for the association between severe dental caries (any tooth with PUFA) prevalence and any impacts on the parent’s life (n = 3879).

Explanatory Variables		%	Unadjusted Associations		Adjusted Associations	
			OR ^a^	[95% CI]	OR ^a^	[95% CI]
*Gender*						
	Male	28.8	1.00	[Reference]	1.00	[Reference]
	Female	30.3	1.07	[0.83–1.38]	1.05	[0.83–1.35]
*Age*						
	5-year-old	21.0	1.00	[Reference]	1.00	[Reference]
	8-year-old	31.8	1.76	[1.29–2.39] ^d^	1.72	[1.28–2.33] ^d^
	12-year-old	30.3	1.64	[1.17–2.30] ^c^	1.79	[1.29–2.46] ^d^
	15-year-old	35.0	2.03	[1.36–3.02] ^c^	2.25	[1.51–3.36] ^d^
*Country*						
	England	29.4	1.00	[Reference]	1.00	[Reference]
	Wales	32.9	1.18	[0.85–1.63]	1.17	[0.84–1.63]
	Northern Ireland	27.2	0.89	[0.73–1.10]	0.91	[0.75–1.11]
*Parent’s marital status*						
	Living with partner	28.6	1.00	[Reference]	1.00	[Reference]
	Living alone	33.0	1.23	[0.98–1.54]	1.13	[0.91–1.42]
*Parent’s education*						
	At degree level or above	29.0	1.00	[Reference]	1.00	[Reference]
	Below degree level	28.3	0.97	[0.69–1.35]	0.90	[0.63–1.26]
	No qualification	37.3	1.46	[0.91–2.33]	1.24	[0.76–2.02]
*Parent’s job classification*						
	Managerial and professional	28.7	1.00	[Reference]	1.00	[Reference]
	Intermediate	25.8	0.87	[0.65–1.15]	0.74	[0.56–0.97] ^b^
	Routine and manual	33.2	1.23	[0.94–1.61]	1.04	[0.77–1.41]
	Never worked	32.0	1.17	[0.81–1.69]	1.00	[0.69–1.45]
*IMD quintiles*						
	Most deprived (Q5)	33.8	1.00	[Reference]	1.00	[Reference]
	Second most deprived (Q4)	32.7	0.95	[0.64–1.43]	1.03	[0.69–1.53]
	Middle (Q3)	26.0	0.69	[0.50–0.96] ^b^	0.79	[0.56–1.11]
	Second least deprived (Q2)	27.3	0.74	[0.50–1.08]	0.78	[0.52–1.17]
	Least deprived (Q1)	25.0	0.65	[0.46–0.93]	0.78	[0.53–1.15]
*Severe dental caries*						
	No teeth with PUFA	28.0	1.00	[Reference]	1.00	[Reference]
	Any tooth with PUFA	66.2	5.03	[2.96–8.55] ^d^	6.00	[3.34–10.78] ^d^

PUFA: pulp involvement, ulceration, fistula, or abscess; IMD: index of multiple deprivation. ^a^ Logistic regression was fitted and adjusted odds ratios (OR) reported. ORs were adjusted for all the variables listed in the table. ^b^ *p* < 0.05, ^c^ *p* < 0.01, ^d^ *p* < 0.001.

**Table 3:** Logistic regression models for the association between severe dental caries (any tooth with PUFA) and specific impacts on the parent’s life (n = 3879).

Severe Dental Caries	%	Unadjusted Associations		Adjusted Associations	
		OR ^a^	[95% CI]	OR ^a^	[95% CI]
Time off work					
No teeth with PUFA	15.1	1.00	[Reference]	1.00	[Reference]
Any tooth with PUFA	25.1	1.88	[0.95–3.72]	2.75	[1.16–6.54] ^b^
Financial difficulties					
No teeth with PUFA	2.4	1.00	[Reference]	1.00	[Reference]
Any tooth with PUFA	3.0	1.27	[0.45–3.62]	1.64	[0.49–5.51]
Child needed more attention					
No teeth with PUFA	12.4	1.00	[Reference]	1.00	[Reference]
Any tooth with PUFA	36.5	4.06	[2.27–7.26] ^d^	4.08	[2.15–7.75] ^d^
Parent’s sleep disrupted					
No teeth with PUFA	6.6	1.00	[Reference]	1.00	[Reference]
Any tooth with PUFA	26.4	5.08	[2.73–9.46] ^d^	4.94	[2.78–8.76] ^d^
Normal activities disrupted					
No teeth with PUFA	5.6	1.00	[Reference]	1.00	[Reference]
Any tooth with PUFA	21.1	4.54	[2.28–9.05] ^d^	5.78	[2.71–12.34] ^d^
Parent felt guilty					
No teeth with PUFA	9.4	1.00	[Reference]	1.00	[Reference]
Any tooth with PUFA	35.7	5.38	[2.94–9.86] ^d^	6.32	[3.26–12.26] ^d^
Parent felt stressed					
No teeth with PUFA	12.8	1.00	[Reference]	1.00	[Reference]
Any tooth with PUFA	49.7	6.73	[4.12–11] ^d^	7.34	[4.15–12.99] ^d^

PUFA: pulp involvement, ulceration, fistula, or abscess; IMD: index of multiple deprivation. ^a^ Logistic regression was fitted and odds ratios (OR) reported. ORs were adjusted for child’s gender, age, and country of residence; parent’s marital status, education, and job classification; and IMD quintiles. ^b^ *p* < 0.05, ^d^ *p* < 0.001.

## References

[1] Kassebaum N.J., Smith A.G.C., Bernabe E., Fleming T.D., Reynolds A.E., Vos T., Murray C.J.L., Marcenes W. (2017). Global, Regional, and National Prevalence, Incidence, and Disability-Adjusted Life Years for Oral Conditions for 195 Countries, 1990–2015: A Systematic Analysis for the Global Burden of Diseases, Injuries, and Risk Factors. J. Dent. Res..

[2] Pitts N.B., Baez R.J., Diaz-Guillory C., Donly K.J., Alberto Feldens C., McGrath C., Phantumvanit P., Seow W.K., Sharkov N., Songpaisan Y. (2019). Early Childhood Caries: IAPD Bangkok Declaration. J. Dent. Child..

[3] Mehta A., Bhalla S. (2014). Assessing consequences of untreated carious lesions using pufa index among 5-6 years old school children in an urban Indian population. Indian J. Dent. Res..

[4] Harris R., Nicoll A.D., Adair P.M., Pine C.M. (2004). Risk factors for dental caries in young children: A systematic review of the literature. Community Dent. Health.

[5] Hooley M., Skouteris H., Boganin C., Satur J., Kilpatrick N. (2012). Parental influence and the development of dental caries in children aged 0–6 years: A systematic review of the literature. J. Dent..

[6] Kim Seow W. (2012). Environmental, maternal, and child factors which contribute to early childhood caries: A unifying conceptual model. Int. J. Paediatr. Dent..

[7] Tinanoff N., Baez R.J., Diaz Guillory C., Donly K.J., Feldens C.A., McGrath C., Phantumvanit P., Pitts N.B., Seow W.K., Sharkov N. (2019). Early childhood caries epidemiology, aetiology, risk assessment, societal burden, management, education, and policy: Global perspective. Int. J. Paediatr. Dent..

[8] Kassebaum N.J., Bernabe E., Dahiya M., Bhandari B., Murray C.J., Marcenes W. (2015). Global burden of untreated caries: A systematic review and metaregression. J. Dent. Res..

[9] World Health Organization (2013). Oral Health Surveys-Basic Methods.

[10] Pitts N.B., Ekstrand K.R. (2013). International Caries Detection and Assessment System (ICDAS) and its International Caries Classification and Management System (ICCMS)-methods for staging of the caries process and enabling dentists to manage caries. Community Dent. Oral Epidemiol..

[11] Monse B., Heinrich-Weltzien R., Benzian H., Holmgren C., van Palenstein Helderman W. (2010). PUFA—An index of clinical consequences of untreated dental caries. Community Dent. Oral Epidemiol..

[12] Holmgren C., van Palenstein Helderman W., Monse B., Heinrich-Weltzien R., Benzian H. (2014). Modifications to the PUFA index: Are they justified at this stage?. Med. Princ. Pract..

[13] Vernazza C.R., Rolland S.L., Chadwick B., Pitts N. (2016). Caries experience, the caries burden and associated factors in children in England, Wales and Northern Ireland 2013. Br. Dent. J..

[14] Bernabe E., Tsakos G., Sheiham A. (2007). Intensity and extent of oral impacts on daily performances by type of self-perceived oral problems. Eur. J. Oral Sci..

[15] Benzian H., Monse B., Heinrich-Weltzien R., Hobdell M., Mulder J., van Palenstein Helderman W. (2011). Untreated severe dental decay: A neglected determinant of low Body Mass Index in 12-year-old Filipino children. BMC Public Health.

[16] Alkarimi H.A., Watt R.G., Pikhart H., Sheiham A., Tsakos G. (2014). Dental caries and growth in school-age children. Pediatrics.

[17] Shen A., Bernabe E., Sabbah W. (2019). Severe dental caries is associated with incidence of thinness and overweight among preschool Chinese children. Acta Odontol. Scand..

[18] Gradella C.M., Bernabe E., Bonecker M., Oliveira L.B. (2011). Caries prevalence and severity, and quality of life in Brazilian 2- to 4-year-old children. Community Dent. Oral Epidemiol..

[19] Krisdapong S., Prasertsom P., Rattanarangsima K., Sheiham A. (2013). School absence due to toothache associated with sociodemographic factors, dental caries status, and oral health-related quality of life in 12- and 15-year-old Thai children. J. Public Health Dent..

[20] Ramos-Jorge J., Pordeus I.A., Ramos-Jorge M.L., Marques L.S., Paiva S.M. (2014). Impact of untreated dental caries on quality of life of preschool children: Different stages and activity. Community Dent. Oral Epidemiol..

[21] Abanto J., Carvalho T.S., Mendes F.M., Wanderley M.T., Bonecker M., Raggio D.P. (2011). Impact of oral diseases and disorders on oral health-related quality of life of preschool children. Community Dent. Oral Epidemiol..

[22] Scarpelli A.C., Paiva S.M., Viegas C.M., Carvalho A.C., Ferreira F.M., Pordeus I.A. (2013). Oral health-related quality of life among Brazilian preschool children. Community Dent. Oral Epidemiol..

[23] Martins-Junior P.A., Vieira-Andrade R.G., Correa-Faria P., Oliveira-Ferreira F., Marques L.S., Ramos-Jorge M.L. (2013). Impact of early childhood caries on the oral health-related quality of life of preschool children and their parents. Caries Res..

[24] Gomes M.C., Pinto-Sarmento T.C., Costa E.M., Martins C.C., Granville-Garcia A.F., Paiva S.M. (2014). Impact of oral health conditions on the quality of life of preschool children and their families: A cross-sectional study. Health Qual. Life Outcomes.

[25] Fernandes I.B., Pereira T.S., Souza D.S., Ramos-Jorge J., Marques L.S., Ramos-Jorge M.L. (2017). Severity of Dental Caries and Quality of Life for Toddlers and Their Families. Pediatr. Dent..

[26] Turton B., Chher T., Sabbah W., Durward C., Hak S., Lailou A. (2019). Epidemiological survey of early childhood caries in Cambodia. BMC Oral Health.

[27] Anderson T., Thomas C., Ryan R., Dennes M., Fuller E. (2015). Children’s Dental Health Survey 2013.

[28] Locker D., Jokovic A., Stephens M., Kenny D., Tompson B., Guyatt G. (2002). Family impact of child oral and oro-facial conditions. Community Dent. Oral Epidemiol..

[29] Jokovic A., Locker D., Stephens M., Kenny D., Tompson B., Guyatt G. (2002). Validity and reliability of a questionnaire for measuring child oral-health-related quality of life. J. Dent. Res..

[30] De Souza M.C., Harrison M., Marshman Z. (2017). Oral health-related quality of life following dental treatment under general anaesthesia for early childhood caries-a UK-based study. Int. J. Paediatr. Dent..

[31] Machry R.V., Tuchtenhagen S., Agostini B.A., da Silva Teixeira C.R., Piovesan C., Mendes F.M., Ardenghi T.M. (2013). Socioeconomic and psychosocial predictors of dental healthcare use among Brazilian preschool children. BMC Oral Health.

[32] Shaban R., Kassim S., Sabbah W. (2017). Socioeconomic inequality in the provision of specific preventive dental interventions among children in the UK: Children’s Dental Health Survey 2003. Br. Dent. J..

[33] Milio N. (1987). Making healthy public policy; developing the science by learning the art: An ecological framework for policy studies. Health Promot. Int..

